# Supplementary material for: Longitudinal impact on rat cardiac tissue transcriptomic profiles due to acute intratracheal inhalation exposures to isoflurane
Source: PLoS One. 2021 Oct 14;16(10):e0257241. doi: 10.1371/journal.pone.0257241 (PMC8516213; doi:10.1371/journal.pone.0257241)
Supplement: S1 Table — Top genes significantly up-regulated between ISO and naive rats at Day 1 are listed. (DOCX) [file pone.0257241.s002.docx]

**S1 Table.**

Top genes increased in hearts by **ISO relative to Naive** on Day 1:

|  | Name | logFC | F | PValue | FDR |
| --- | --- | --- | --- | --- | --- |
| 1 | Ephx1 | 0.93 | 53.46 | 6.00e-07 | 7.72e-03 |
| 2 | Cep128 | 0.63 | 37.26 | 6.00e-06 | 1.98e-02 |
| 3 | Ptgds | 1.87 | 40.54 | 6.70e-06 | 1.98e-02 |
| 4 | Bnip3 | 0.65 | 35.84 | 8.40e-06 | 1.98e-02 |
| 5 | Myoz2 | 0.44 | 34.10 | 1.06e-05 | 1.98e-02 |
| 6 | AABR07067600.1 | 0.61 | 32.00 | 1.75e-05 | 2.39e-02 |
| 7 | Mt-nd6 | 0.63 | 30.23 | 2.26e-05 | 2.51e-02 |
| 8 | Slc25a33 | 0.41 | 29.45 | 2.65e-05 | 2.51e-02 |
| 9 | Cry2 | 0.53 | 28.78 | 3.16e-05 | 2.51e-02 |
| 10 | Mt-nd5 | 0.59 | 28.21 | 3.44e-05 | 2.51e-02 |
| 11 | Lonrf1 | 0.83 | 28.13 | 3.50e-05 | 2.51e-02 |
| 12 | Irs2 | 0.64 | 27.21 | 4.92e-05 | 3.11e-02 |
| 13 | Htr2a | 0.67 | 26.88 | 5.73e-05 | 3.12e-02 |
| 14 | Sccpdh | 0.74 | 26.85 | 5.95e-05 | 3.12e-02 |
| 15 | Per2 | 1.07 | 25.72 | 9.58e-05 | 4.16e-02 |
| 16 | Gadd45b | 0.71 | 22.88 | 1.18e-04 | 4.30e-02 |
| 17 | Cd63 | 0.42 | 22.58 | 1.24e-04 | 4.30e-02 |
| 18 | Nqo1 | 0.40 | 22.49 | 1.26e-04 | 4.30e-02 |
| 19 | AY172581.20 | 0.72 | 22.80 | 1.50e-04 | 4.83e-02 |
| 20 | Nanp | 0.60 | 21.30 | 1.70e-04 | 5.02e-02 |
| 21 | RGD1310507 | 0.43 | 21.17 | 1.76e-04 | 5.05e-02 |
| 22 | AY172581.23 | 0.73 | 20.14 | 2.28e-04 | 5.84e-02 |
| 23 | Cd200 | 0.45 | 20.13 | 2.32e-04 | 5.84e-02 |
| 24 | Lrtm2 | 1.79 | 19.81 | 2.57e-04 | 6.16e-02 |
| 25 | Nrap | 0.47 | 19.24 | 2.95e-04 | 6.16e-02 |
| 26 | LOC103691744_1 | 1.04 | 19.10 | 3.00e-04 | 6.16e-02 |
| 27 | Rgcc | 0.72 | 20.12 | 3.02e-04 | 6.16e-02 |
| 28 | LOC100911766 | 0.68 | 19.34 | 3.41e-04 | 6.16e-02 |
| 29 | Dgat2 | 0.62 | 18.95 | 3.58e-04 | 6.16e-02 |
| 30 | Tef | 0.79 | 19.12 | 4.04e-04 | 6.63e-02 |
