# Supplementary material for: Longitudinal impact on rat cardiac tissue transcriptomic profiles due to acute intratracheal inhalation exposures to isoflurane
Source: PLoS One. 2021 Oct 14;16(10):e0257241. doi: 10.1371/journal.pone.0257241 (PMC8516213; doi:10.1371/journal.pone.0257241)
Supplement: S2 Table — Top genes significantly up-regulated between ISO and naive rats at Day 30 are listed. (DOCX) [file pone.0257241.s003.docx]

**S2 Table.**

Top genes increased in hearts by **ISO relative to Naive** on Day 30:

|  | Name | logFC | F | PValue | FDR |
| --- | --- | --- | --- | --- | --- |
| 1 | Rn50_19_0291.1 | 1.54 | 31.65 | 1.60e-05 | 4.08e-02 |
| 2 | Abca1 | 0.66 | 19.09 | 2.93e-04 | 2.65e-01 |
| 3 | AABR07019085.1 | 1.36 | 17.85 | 4.20e-04 | 2.85e-01 |
| 4 | Fat4 | 0.84 | 14.39 | 1.17e-03 | 3.98e-01 |
| 5 | Lifr | 0.66 | 14.62 | 1.27e-03 | 4.07e-01 |
| 6 | Sesn3 | 0.56 | 13.55 | 1.38e-03 | 4.07e-01 |
| 7 | AABR07033887.1 | 1.26 | 14.87 | 1.39e-03 | 4.07e-01 |
| 8 | C4b | 0.98 | 13.92 | 1.43e-03 | 4.07e-01 |
| 9 | RGD1561897 | 0.63 | 13.23 | 1.44e-03 | 4.07e-01 |
| 10 | Il18 | 0.87 | 13.16 | 1.47e-03 | 4.08e-01 |
| 11 | Slco5a1 | 0.55 | 12.22 | 2.05e-03 | 4.79e-01 |
| 12 | Cep350 | 0.50 | 12.40 | 2.08e-03 | 4.79e-01 |
| 13 | Clstn2 | 0.53 | 12.19 | 2.25e-03 | 4.90e-01 |
| 14 | Zfp871 | 0.84 | 11.66 | 2.46e-03 | 4.91e-01 |
| 15 | Zyg11b | 0.69 | 12.25 | 2.55e-03 | 4.91e-01 |
| 16 | Med12l | 0.56 | 11.44 | 2.66e-03 | 5.01e-01 |
| 17 | Ptprb | 0.53 | 11.99 | 3.01e-03 | 5.21e-01 |
| 18 | Mfap3l | 0.51 | 11.14 | 3.03e-03 | 5.21e-01 |
| 19 | LOC100911825 | 0.78 | 10.81 | 3.33e-03 | 5.36e-01 |
| 20 | Cd180 | 0.85 | 10.79 | 3.35e-03 | 5.36e-01 |
| 21 | Arl11 | 0.86 | 10.51 | 3.72e-03 | 5.44e-01 |
| 22 | Nt5c | 0.62 | 10.21 | 4.15e-03 | 5.44e-01 |
| 23 | Cxcl9 | 0.45 | 10.20 | 4.16e-03 | 5.44e-01 |
| 24 | Uhmk1 | 0.45 | 10.17 | 4.22e-03 | 5.44e-01 |
| 25 | Birc6 | 0.64 | 10.82 | 4.25e-03 | 5.44e-01 |
| 26 | Cd163 | 0.77 | 10.46 | 4.45e-03 | 5.44e-01 |
| 27 | ENSRNOG00000051570 | 0.59 | 9.98 | 4.51e-03 | 5.44e-01 |
| 28 | Zfp106 | 0.51 | 10.74 | 4.55e-03 | 5.44e-01 |
| 29 | Rn60_9_0652.2 | 0.53 | 9.93 | 4.60e-03 | 5.44e-01 |
| 30 | Nxt2 | 0.57 | 9.86 | 4.73e-03 | 5.44e-01 |
