# Supplementary material for: Longitudinal impact on rat cardiac tissue transcriptomic profiles due to acute intratracheal inhalation exposures to isoflurane
Source: PLoS One. 2021 Oct 14;16(10):e0257241. doi: 10.1371/journal.pone.0257241 (PMC8516213; doi:10.1371/journal.pone.0257241)
Supplement: S3 Table — Top genes significantly up-regulated between ISO and naive rats at Day 240 are listed. (DOCX) [file pone.0257241.s004.docx]

**S3 Table.**

Top genes increased in hearts by **ISO relative to Naive** on Day 240:

|  | Name | logFC | F | PValue | FDR |
| --- | --- | --- | --- | --- | --- |
| 1 | Arhgef15 | 0.35 | 32.18 | 1.78e-05 | 1.38e-01 |
| 2 | Notch1 | 0.45 | 24.46 | 8.84e-05 | 1.38e-01 |
| 3 | Mbd6 | 0.34 | 21.99 | 1.58e-04 | 1.38e-01 |
| 4 | Notch4 | 0.58 | 19.44 | 2.98e-04 | 1.39e-01 |
| 5 | Cntrob | 0.67 | 18.64 | 3.67e-04 | 1.44e-01 |
| 6 | Dusp26 | 0.40 | 15.39 | 9.05e-04 | 1.66e-01 |
| 7 | Slc4a1 | 0.49 | 14.94 | 1.03e-03 | 1.66e-01 |
| 8 | Ddb2 | 0.38 | 14.35 | 1.23e-03 | 1.73e-01 |
| 9 | Tgm4 | 0.39 | 13.58 | 1.56e-03 | 1.73e-01 |
| 10 | LOC100912262 | 0.47 | 13.43 | 1.63e-03 | 1.75e-01 |
| 11 | Helz2 | 0.43 | 13.23 | 1.74e-03 | 1.82e-01 |
| 12 | Gja5 | 0.34 | 12.54 | 2.17e-03 | 1.98e-01 |
| 13 | Prx | 0.44 | 12.47 | 2.22e-03 | 1.99e-01 |
| 14 | Bcl6b | 0.37 | 12.41 | 2.26e-03 | 1.99e-01 |
| 15 | Zc3h3 | 0.34 | 11.96 | 2.62e-03 | 2.19e-01 |
| 16 | Cbx2 | 0.58 | 11.80 | 2.76e-03 | 2.22e-01 |
| 17 | Zswim8 | 0.43 | 11.62 | 2.92e-03 | 2.25e-01 |
| 18 | Map10 | 0.38 | 11.37 | 3.18e-03 | 2.26e-01 |
| 19 | Tmie | 0.62 | 11.32 | 3.23e-03 | 2.26e-01 |
| 20 | Rtp3 | 0.65 | 11.27 | 3.30e-03 | 2.26e-01 |
| 21 | Cbfa2t3 | 0.39 | 11.25 | 3.31e-03 | 2.26e-01 |
| 22 | Fryl | 0.33 | 11.06 | 3.53e-03 | 2.35e-01 |
| 23 | Cfap70 | 0.34 | 11.03 | 3.57e-03 | 2.36e-01 |
| 24 | Coro2b | 0.34 | 10.81 | 3.85e-03 | 2.37e-01 |
| 25 | Nid2 | 0.38 | 10.69 | 4.02e-03 | 2.37e-01 |
| 26 | Daam2 | 0.45 | 10.61 | 4.13e-03 | 2.39e-01 |
| 27 | Asap2 | 0.39 | 10.44 | 4.37e-03 | 2.42e-01 |
| 28 | Ccdc157 | 0.43 | 10.43 | 4.40e-03 | 2.42e-01 |
| 29 | Bean1 | 0.45 | 10.41 | 4.41e-03 | 2.42e-01 |
| 30 | Ccdc120 | 0.56 | 10.11 | 4.91e-03 | 2.54e-01 |
