# Supplementary material for: Longitudinal impact on rat cardiac tissue transcriptomic profiles due to acute intratracheal inhalation exposures to isoflurane
Source: PLoS One. 2021 Oct 14;16(10):e0257241. doi: 10.1371/journal.pone.0257241 (PMC8516213; doi:10.1371/journal.pone.0257241)
Supplement: S4 Table — Top genes significantly up-regulated between ISO and naive rats at Day 360 are listed. (DOCX) [file pone.0257241.s005.docx]

**S4 Table.**

Top genes increased in hearts by **ISO relative to Naive** on Day 360:

|  | Name | logFC | F | PValue | FDR |
| --- | --- | --- | --- | --- | --- |
| 1 | Sphk1 | 0.86 | 77.54 | 2.05e-08 | 8.97e-05 |
| 2 | Ssc5d | 1.01 | 70.13 | 4.64e-08 | 1.52e-04 |
| 3 | Bdkrb2 | 1.33 | 58.73 | 1.88e-07 | 2.54e-04 |
| 4 | Il1r1 | 0.81 | 58.47 | 1.95e-07 | 2.54e-04 |
| 5 | Ncam1 | 1.07 | 61.40 | 2.54e-07 | 2.54e-04 |
| 6 | Adamtsl2 | 1.38 | 56.45 | 3.55e-07 | 2.54e-04 |
| 7 | Phyhipl | 1.56 | 53.86 | 3.65e-07 | 2.54e-04 |
| 8 | Ltbp2 | 1.98 | 62.14 | 5.06e-07 | 2.82e-04 |
| 9 | Plaur | 1.07 | 50.86 | 5.61e-07 | 2.83e-04 |
| 10 | C1qtnf5 | 0.92 | 47.79 | 8.87e-07 | 3.76e-04 |
| 11 | Fam167a | 1.50 | 47.76 | 9.24e-07 | 3.79e-04 |
| 12 | Ankrd23 | 0.78 | 49.27 | 1.02e-06 | 3.81e-04 |
| 13 | Adamts8 | 1.22 | 46.29 | 1.16e-06 | 4.08e-04 |
| 14 | Col12a1 | 0.80 | 45.42 | 1.28e-06 | 4.16e-04 |
| 15 | Fjx1 | 1.11 | 43.01 | 1.90e-06 | 5.43e-04 |
| 16 | Meox1 | 1.31 | 42.99 | 1.90e-06 | 5.43e-04 |
| 17 | Col8a1 | 1.20 | 44.74 | 2.14e-06 | 5.51e-04 |
| 18 | Loxl1 | 1.23 | 46.81 | 2.38e-06 | 5.83e-04 |
| 19 | Cilp | 1.50 | 45.36 | 2.46e-06 | 5.83e-04 |
| 20 | Crtac1 | 1.43 | 41.29 | 2.53e-06 | 5.83e-04 |
| 21 | Ctsk | 0.81 | 41.58 | 2.64e-06 | 5.83e-04 |
| 22 | Nid2 | 1.04 | 40.38 | 3.15e-06 | 6.33e-04 |
| 23 | Thbs4 | 2.06 | 46.82 | 3.95e-06 | 6.74e-04 |
| 24 | Tmem100 | 0.95 | 38.98 | 4.22e-06 | 6.84e-04 |
| 25 | Nppa | 1.45 | 42.61 | 4.54e-06 | 7.18e-04 |
| 26 | Tgfb1i1 | 0.78 | 38.14 | 4.95e-06 | 7.52e-04 |
| 27 | Hmcn2 | 0.79 | 39.62 | 4.98e-06 | 7.52e-04 |
| 28 | Tgfb2 | 0.99 | 39.41 | 6.61e-06 | 8.58e-04 |
| 29 | Pcsk5 | 0.82 | 35.29 | 7.39e-06 | 8.96e-04 |
| 30 | Col1a1 | 1.30 | 40.44 | 7.58e-06 | 9.04e-04 |
