# Supplementary material for: Longitudinal impact on rat cardiac tissue transcriptomic profiles due to acute intratracheal inhalation exposures to isoflurane
Source: PLoS One. 2021 Oct 14;16(10):e0257241. doi: 10.1371/journal.pone.0257241 (PMC8516213; doi:10.1371/journal.pone.0257241)
Supplement: S5 Table — Top genes significantly down-regulated between ISO and naive rats at Day 1 are listed. (DOCX) [file pone.0257241.s006.docx]

**S5 Table.**

Top genes reduced in hearts by **ISO relative to Naive** on Day 1:

|  | Name | logFC | F | PValue | FDR |
| --- | --- | --- | --- | --- | --- |
| 1 | Racgap1 | -0.88 | 32.02 | 1.58e-05 | 2.39e-02 |
| 2 | Nek6 | -0.80 | 25.27 | 6.57e-05 | 3.20e-02 |
| 3 | Aurkb | -1.24 | 23.54 | 9.82e-05 | 4.16e-02 |
| 4 | Npas2 | -1.44 | 22.63 | 1.78e-04 | 5.05e-02 |
| 5 | LOC100359539 | -1.21 | 20.94 | 1.86e-04 | 5.16e-02 |
| 6 | Kifc1 | -1.05 | 20.79 | 1.93e-04 | 5.16e-02 |
| 7 | Spon2_1 | -1.04 | 18.60 | 3.43e-04 | 6.16e-02 |
| 8 | Kif23 | -1.15 | 18.49 | 3.53e-04 | 6.16e-02 |
| 9 | Kif20a | -1.46 | 18.51 | 3.54e-04 | 6.16e-02 |
| 10 | Bub1b | -1.19 | 18.13 | 3.90e-04 | 6.55e-02 |
| 11 | Cenpe | -0.80 | 17.10 | 5.19e-04 | 7.74e-02 |
| 12 | Ncaph | -1.20 | 16.37 | 6.39e-04 | 9.26e-02 |
| 13 | Ckap2 | -1.06 | 16.31 | 6.50e-04 | 9.32e-02 |
| 14 | Ckap2l | -1.52 | 15.82 | 7.49e-04 | 9.53e-02 |
| 15 | Arntl | -1.93 | 16.04 | 1.04e-03 | 1.10e-01 |
| 16 | Evi2a | -1.33 | 14.79 | 1.04e-03 | 1.10e-01 |
| 17 | Gas2l3 | -0.92 | 14.71 | 1.04e-03 | 1.10e-01 |
| 18 | Dlgap5 | -0.76 | 14.47 | 1.12e-03 | 1.12e-01 |
| 19 | Cacna1h | -1.51 | 15.21 | 1.24e-03 | 1.19e-01 |
| 20 | Cdk1 | -1.16 | 14.13 | 1.24e-03 | 1.19e-01 |
| 21 | Iqgap3 | -1.10 | 14.19 | 1.25e-03 | 1.19e-01 |
| 22 | Myl7 | -9.95 | 15.52 | 1.27e-03 | 1.19e-01 |
| 23 | Cxcl13 | -1.47 | 13.82 | 1.46e-03 | 1.25e-01 |
| 24 | LOC100911825 | -0.87 | 13.51 | 1.51e-03 | 1.26e-01 |
| 25 | Tnfrsf10b | -0.71 | 13.48 | 1.52e-03 | 1.26e-01 |
| 26 | Fcgbp | -0.79 | 13.23 | 1.65e-03 | 1.32e-01 |
| 27 | Plk1 | -0.87 | 13.17 | 1.69e-03 | 1.32e-01 |
| 28 | Col5a3 | -1.12 | 13.96 | 1.72e-03 | 1.32e-01 |
| 29 | Pitpnm3 | -0.88 | 12.80 | 1.90e-03 | 1.34e-01 |
| 30 | Cdca8 | -0.72 | 12.71 | 1.96e-03 | 1.36e-01 |
