# Supplementary material for: Longitudinal impact on rat cardiac tissue transcriptomic profiles due to acute intratracheal inhalation exposures to isoflurane
Source: PLoS One. 2021 Oct 14;16(10):e0257241. doi: 10.1371/journal.pone.0257241 (PMC8516213; doi:10.1371/journal.pone.0257241)
Supplement: S6 Table — Top genes significantly down-regulated between ISO and naive rats at Day 30 are listed. (DOCX) [file pone.0257241.s007.docx]

**S6 Table.**

Top genes reduced in hearts by **ISO relative to Naive** on Day 30:

|  | Name | logFC | F | PValue | FDR |
| --- | --- | --- | --- | --- | --- |
| 1 | RGD1305298 | -0.38 | 31.91 | 1.08e-05 | 4.08e-02 |
| 2 | AABR07063424.1 | -1.20 | 38.59 | 1.29e-05 | 4.08e-02 |
| 3 | AABR07015055.1 | -1.19 | 37.27 | 1.58e-05 | 4.08e-02 |
| 4 | Abra | -0.37 | 29.51 | 1.81e-05 | 4.08e-02 |
| 5 | AABR07015078.1 | -1.16 | 35.51 | 2.08e-05 | 4.08e-02 |
| 6 | AABR07015066.1 | -1.17 | 35.49 | 2.10e-05 | 4.08e-02 |
| 7 | AABR07015080.2 | -1.16 | 34.51 | 2.46e-05 | 4.18e-02 |
| 8 | AABR07000398.1 | -1.05 | 30.89 | 4.17e-05 | 5.78e-02 |
| 9 | LOC257642_1 | -1.10 | 31.10 | 4.26e-05 | 5.78e-02 |
| 10 | LOC102553613 | -1.20 | 21.84 | 1.14e-04 | 1.29e-01 |
| 11 | Slc38a4 | -0.71 | 17.80 | 3.47e-04 | 2.78e-01 |
| 12 | LOC257642_3 | -1.11 | 18.07 | 6.50e-04 | 3.48e-01 |
| 13 | LOC100910478 | -0.62 | 14.86 | 8.47e-04 | 3.63e-01 |
| 14 | AABR07060133.1 | -0.87 | 14.16 | 1.06e-03 | 3.79e-01 |
| 15 | Nmnat1 | -0.44 | 13.11 | 1.60e-03 | 4.34e-01 |
| 16 | Inha | -0.44 | 12.30 | 1.97e-03 | 4.76e-01 |
| 17 | Cntnap2 | -0.45 | 11.95 | 2.22e-03 | 4.90e-01 |
| 18 | Grm8 | -0.76 | 11.07 | 3.03e-03 | 5.21e-01 |
| 19 | Rpl10a | -0.47 | 10.95 | 3.16e-03 | 5.30e-01 |
| 20 | Cat | -0.44 | 10.89 | 4.13e-03 | 5.44e-01 |
| 21 | AABR07035916.1 | -0.66 | 10.11 | 4.31e-03 | 5.44e-01 |
| 22 | Qtrt1 | -0.42 | 10.10 | 4.31e-03 | 5.44e-01 |
| 23 | Lrrtm3 | -0.53 | 10.07 | 4.36e-03 | 5.44e-01 |
| 24 | AABR07031150.1 | -0.43 | 9.89 | 4.66e-03 | 5.44e-01 |
| 25 | St6galnac3 | -0.41 | 9.77 | 4.89e-03 | 5.58e-01 |
| 26 | Spc24 | -0.67 | 9.23 | 5.99e-03 | 5.85e-01 |
| 27 | Ddit3 | -0.49 | 9.75 | 6.21e-03 | 5.93e-01 |
| 28 | Atg4a | -0.38 | 9.02 | 6.53e-03 | 5.98e-01 |
| 29 | Med18 | -0.49 | 8.82 | 7.02e-03 | 6.08e-01 |
| 30 | Hpn | -0.48 | 8.72 | 7.30e-03 | 6.08e-01 |
