# Supplementary material for: Longitudinal impact on rat cardiac tissue transcriptomic profiles due to acute intratracheal inhalation exposures to isoflurane
Source: PLoS One. 2021 Oct 14;16(10):e0257241. doi: 10.1371/journal.pone.0257241 (PMC8516213; doi:10.1371/journal.pone.0257241)
Supplement: S7 Table — Top genes significantly down-regulated between ISO and naive rats at Day 240 are listed. (DOCX) [file pone.0257241.s008.docx]

**S7 Table.**

Top genes reduced in hearts by **ISO relative to Naive** on Day 240:

|  | Name | logFC | F | PValue | FDR |
| --- | --- | --- | --- | --- | --- |
| 1 | Nr4a1 | -1.65 | 26.92 | 5.15e-05 | 1.38e-01 |
| 2 | C4b | -1.43 | 21.98 | 1.58e-04 | 1.38e-01 |
| 3 | Usmg5 | -0.52 | 21.92 | 1.60e-04 | 1.38e-01 |
| 4 | Nr4a3 | -2.82 | 22.02 | 1.67e-04 | 1.38e-01 |
| 5 | S100a4 | -0.80 | 21.15 | 1.93e-04 | 1.38e-01 |
| 6 | Rpl22l1 | -0.67 | 20.41 | 2.33e-04 | 1.38e-01 |
| 7 | Rassf2 | -0.70 | 19.78 | 2.73e-04 | 1.38e-01 |
| 8 | Atf3 | -1.12 | 19.73 | 2.76e-04 | 1.38e-01 |
| 9 | Dmbt1 | -1.00 | 19.42 | 3.00e-04 | 1.39e-01 |
| 10 | Slc11a1 | -0.78 | 16.80 | 6.06e-04 | 1.66e-01 |
| 11 | AC110709.1 | -0.50 | 15.64 | 8.43e-04 | 1.66e-01 |
| 12 | Kcnu1 | -0.71 | 15.48 | 8.82e-04 | 1.66e-01 |
| 13 | Spp1 | -2.35 | 15.65 | 8.90e-04 | 1.66e-01 |
| 14 | Rpl22l2 | -0.65 | 15.37 | 9.10e-04 | 1.66e-01 |
| 15 | Apoe | -0.71 | 15.23 | 9.49e-04 | 1.66e-01 |
| 16 | Rps25 | -0.55 | 15.17 | 9.67e-04 | 1.66e-01 |
| 17 | AC126572.3 | -0.56 | 15.16 | 9.68e-04 | 1.66e-01 |
| 18 | Igf1 | -0.56 | 15.02 | 1.01e-03 | 1.66e-01 |
| 19 | Plvap | -1.09 | 14.71 | 1.11e-03 | 1.69e-01 |
| 20 | Fcgr2b | -1.07 | 14.17 | 1.30e-03 | 1.73e-01 |
| 21 | Serpinb1a | -1.21 | 14.14 | 1.31e-03 | 1.73e-01 |
| 22 | Ccl6 | -0.82 | 14.04 | 1.36e-03 | 1.73e-01 |
| 23 | Defb1 | -1.01 | 13.87 | 1.43e-03 | 1.73e-01 |
| 24 | Lyve1 | -0.95 | 13.87 | 1.43e-03 | 1.73e-01 |
| 25 | Cfd | -0.88 | 13.37 | 1.66e-03 | 1.77e-01 |
| 26 | Btg2 | -0.77 | 13.08 | 1.82e-03 | 1.85e-01 |
| 27 | Fgl2 | -0.68 | 12.79 | 2.00e-03 | 1.92e-01 |
| 28 | Slamf9 | -0.88 | 12.78 | 2.01e-03 | 1.92e-01 |
| 29 | Cebpa | -0.71 | 12.68 | 2.07e-03 | 1.93e-01 |
| 30 | Slc22a1 | -0.50 | 12.61 | 2.12e-03 | 1.95e-01 |
