# Supplementary material for: Longitudinal impact on rat cardiac tissue transcriptomic profiles due to acute intratracheal inhalation exposures to isoflurane
Source: PLoS One. 2021 Oct 14;16(10):e0257241. doi: 10.1371/journal.pone.0257241 (PMC8516213; doi:10.1371/journal.pone.0257241)
Supplement: S8 Table — Top genes significantly down-regulated between ISO and naive rats at Day 360 are listed. (DOCX) [file pone.0257241.s009.docx]

**S8 Table.**

Top genes reduced in hearts by **ISO relative to Naive** on Day 360:

|  | Name | logFC | F | PValue | FDR |
| --- | --- | --- | --- | --- | --- |
| 1 | Fitm2 | -0.60 | 61.16 | 1.00e-07 | 2.54e-04 |
| 2 | Gsta4 | -0.57 | 59.29 | 2.00e-07 | 2.54e-04 |
| 3 | Piwil2 | -0.67 | 53.21 | 4.00e-07 | 2.54e-04 |
| 4 | AABR07010868.1 | -0.58 | 52.87 | 4.00e-07 | 2.54e-04 |
| 5 | Ddit4l | -0.82 | 52.76 | 4.00e-07 | 2.54e-04 |
| 6 | LOC103689947 | -0.57 | 51.22 | 5.00e-07 | 2.82e-04 |
| 7 | Eci1 | -0.54 | 50.23 | 6.00e-07 | 2.99e-04 |
| 8 | Slc22a3 | -0.52 | 45.16 | 1.30e-06 | 4.18e-04 |
| 9 | Ech1 | -0.62 | 41.25 | 2.50e-06 | 5.83e-04 |
| 10 | Rab3b | -0.58 | 41.44 | 2.60e-06 | 5.83e-04 |
| 11 | Dhrs7c | -0.55 | 40.84 | 2.70e-06 | 5.83e-04 |
| 12 | Acot1 | -1.15 | 37.45 | 5.00e-06 | 7.52e-04 |
| 13 | Atp5e | -0.51 | 37.12 | 5.30e-06 | 7.57e-04 |
| 14 | Lsamp | -0.66 | 37.08 | 5.30e-06 | 7.57e-04 |
| 15 | Ros1 | -0.61 | 33.98 | 9.50e-06 | 1.00e-03 |
| 16 | Kcnj5 | -0.54 | 33.76 | 1.00e-05 | 1.00e-03 |
| 17 | Kcnu1 | -0.71 | 33.31 | 1.08e-05 | 1.03e-03 |
| 18 | AABR07027872.1 | -1.14 | 33.13 | 1.12e-05 | 1.04e-03 |
| 19 | Carmil3 | -0.59 | 32.96 | 1.16e-05 | 1.06e-03 |
| 20 | Cyp2e1 | -1.92 | 34.33 | 1.86e-05 | 1.30e-03 |
| 21 | Gstm7 | -0.71 | 31.93 | 1.89e-05 | 1.31e-03 |
| 22 | Perp | -0.56 | 30.82 | 2.06e-05 | 1.38e-03 |
| 23 | Pdxp | -0.52 | 29.84 | 2.18e-05 | 1.39e-03 |
| 24 | Tmod4 | -0.58 | 29.44 | 2.38e-05 | 1.44e-03 |
| 25 | Icoslg | -0.63 | 29.40 | 2.40e-05 | 1.44e-03 |
| 26 | Rdm1 | -0.51 | 29.39 | 2.40e-05 | 1.44e-03 |
| 27 | Phkg1 | -0.69 | 29.21 | 2.50e-05 | 1.46e-03 |
| 28 | Dcxr | -0.64 | 30.04 | 2.55e-05 | 1.48e-03 |
| 29 | Slc27a1 | -0.55 | 29.36 | 2.82e-05 | 1.58e-03 |
| 30 | Rpl22l1 | -0.57 | 28.51 | 3.33e-05 | 1.69e-03 |
