# Supplementary material for: Longitudinal impact on rat cardiac tissue transcriptomic profiles due to acute intratracheal inhalation exposures to isoflurane
Source: PLoS One. 2021 Oct 14;16(10):e0257241. doi: 10.1371/journal.pone.0257241 (PMC8516213; doi:10.1371/journal.pone.0257241)
Supplement: Attachment — Submitted filename: Response to Reviewers.pdf [file pone.0257241.s011.pdf]

PONE-D-21-09743
Longitudinal Impact on Rat Cardiac Tissue Transcriptomic Profiles due to Acute Intra-tracheal Inhalation Exposures to Isoflurane

Dear Dr. Park,
Thank you for submitting your manuscript to PLOS ONE. After careful consideration, we feel that it has merit but does not fully meet PLOS ONE's publication criteria as it currently stands. Therefore, we invite you to submit a revised version of the manuscript that addresses the points raised during the review process.

Two expert reviewers have studied your manuscript. Although both find the topic of interest they have made several comments that you will need to address in a revised manuscript. Particularly, reviewer #1 has identified flaws in your experimental design that can only be repaired by including additional groups. Hence, experiments should be re-done in SHR rats receiving free, spontaneous inhalation with ISO compared to air only. In addition, control WKY rats receiving free, spontaneous inhalation with ISO compared to air only, should also be included. However, I find it acceptable if these additional groups are not followed for 240 and 360 days. The earlier timepoints (D1 and D30) will suffice.

If you will need more time than 6 mo to complete your revisions, please reply to this message or contact the journal office at plosone@plos.org. When you're ready to submit your revision, log on to https://www.editorialmanager.com/pone/ and select the 'Submissions Needing Revision' folder to locate your manuscript file.

Response: As we note in the Response to Reviewers' Comments, in the context of our ongoing WTC studies of heart disease in First Responders exposed at Ground Zero, at this point in 2021, the outcomes at the early post-exposure timepoints here are of less import than the longer-term ones. As a result, any experiment in WKY rats (with/without ITIH) or spontaneous SHR ISO rats would of necessity need to measure effects at Day 360 post-exposures. That would take ≈ 1.5 yr to complete the exposures and RNA analyses; so, no revision would be ready in < 1.5-2 years.

The EIC offer to only look at very short post-exposure timepoints is very generous. However, we have decided we will not perform any new WKY (with/without ITIH) or spontaneous SHR ISO studies. Justification for this is provided below. If the EIC deems it the best way to move the MS forward in light of the concerns about potential confounding by an Insyte tube, we will be more than ready to eliminate the early post-exposure timepoint data (Day 1 and Day 30)/related discussion from the MS and solely focus on the longer-term implications from the ISO exposures.

Accordingly, until we hear else-wise, we are submitting a revised MS that addresses all the other 'non-Insyte' comments of the Reviewers.

Reviewer #1
Aim of the study described in Park et al. is to elucidate effect of inhalation anesthetic isoflurane (ISO) on gene expression patterns of cardiac tissue. While ISO may not lead to long-lasting side-effects, short-\long-term effects on gene regulation remain unexplored. Park et al. investigated the role of ISO on SHR rats by 2 consecutive days of 2-hr ISO (2%) intratracheal inhalation (IHIH)

vs. air only inhalation. Hearts were explanted on D1, D30, D240 and D360 post-exposure. Their main finding was that ISO exposure leads to changes in cardiac gene expression, some of which last up until D360. The primary pathways involved included general immune response e.g., wound healing and cytokine-mediated pathways, circadian rhythm, regulation of systemic arterial blood pressure, fatty acid metabolism and extracellular matrix organization.

I appreciate the effort put into this MS as indeed inhalation anesthetics, often used in performing experimental cardiovascular surgeries on rodents, may unintentionally influence (cardiac) cellular processes. However, in most of these cases, both controls/Shams and experimental intervention groups will receive the same (type of) anesthetic treatment. In this MS, the methods describe that control animals did not receive ITIH. The real comparison here is thus SHR + spontaneous breathing vs. SHR + mechanical breathing on ISO. I believe it is thus not possible to discern between the effects of ISO vs ITIH on cardiac gene expression and thus impossible to attribute the findings to ISO alone.

Response: We appreciate the Reviewer's concern about what would constitute a 'proper' comparative control. As alluded to below and in some of Reviewer #2's comments, the concern is that the 'instilled' tube itself (a rounded-off tip of a 18G Insyte) is somehow causative of the responses noted here.

There seem two ways to try to potentially address the concern: (1) intubate rats 2 hr/2 days while under some other anesthetic during ITIH to see if 'same' effects are seen, thereby implicating the Insyte tube, or (2) place rats in a chamber to allow for 2 hr nose-only breathing of ISO (starting at 5% and then maintained at 2.5%) to see if the tube caused ALL the observed effects here. The first approach is ruled out as there would be no way to control for anesthetic-specific effects that would hamper comparisons of results (refer to multiple papers that compare ISO vs. sevoflurane vs. desflurane inhalants) and so would not allow tube-specific effects to be discerned clearly. The second approach, while ultimately do-able, would basically reiterate findings from a paper by DeLano and Zeifach (1981) that noted some changes in systemic blood pressure in SHR rats (vs. WKY rats) after anesthesia (not ISO) and surgical intubation (open trachea and then insert tube; not method here). However, any effects from the intubation were short-lived and decreased over the period of time the tube was present (within hours). The authors could not explain the transient pressor effect in the SHR rats and noted that any similar effect in humans was mitigated by coating the tube with lidocaine. A more recent study (Konno et al. 2014) found endotracheal intubation of rats under ISO (and then maintained under ISO) for periods beyond 30 min had no impact on vital signs and caused no histopathological trauma to the lungs or the trachea.

Thus, while we might concede that some of the reported outcomes here at Day 1 (i.e., wound-healing pathways activated, inflammation) could potentially be tied back to presence of the tube for those two 2-hr periods, those expression changes were gone by > Day 30 post-exposure and did not return. We respectfully do not concede the very short presence of a tube in the upper trachea of each rat was what led to the outcomes in the heart at Days 240 and 360 post-exposure.

The choice to use non-intubated controls was well-thought out and recommended/approved by the Study Section experts who reviewed/modified the original grant. After discussing this particular Reviewers' concern with experts in Inhalation Toxicology and in animal model use, it was agreed

the approach used allowed us to clearly deduce the longer-term effects specifically induced by ISO here. Accordingly, if both Reviewers think the data for the earliest post-exposure timepoints (Day 1, 30) remain problematic, we would consider just having the paper focus on the longer-term impact of the two ISO exposures. This, in fact, would be of more relevance to our original study, i.e., to understand potential effects on hearts of First Responders exposed to World Trade Center dusts, using the SHR model. In that context (to explain why FR have/still manifest elevated incidences of cardiovascular disorders even 20 years after the disaster), the Day 1 and Day 30 ISO data ultimately become of little import.

In the end, ISO effects on gene expression in the hearts of our SHR rats were unexpected; even less expected were the changing patterns of expression seen as post-exposure period progressed. Thus, we believed - and still do - that sharing this novel unexpected information about potential short-/long-term implications from ISO use to clinicians who routinely employ ISO: for the long-term in sedation of critically-ill children; for prolonged periods during post-operative intensive care of some cardiac surgery patients; and, potentially in long-term critical care sedation (VALTS) of ICU patients, could prove useful in their deciding if continued use of ISO in these cases is appropriate in the end.

Please find a more detailed overview of my comments below.
Major:
1) Regarding experimental set-up: From the MS it becomes clear that ISO but not the control group received ITIH. There is evidence that mechanical ventilation affects cardiovascular functioning (e.g. PMID: 2199000, PMID: 10208959). So how sure are the Authors that the induced changes in gene expression are due to ISO and not the ITIH? I think the fact that for instance the wound-healing pathways are activated, says more about the effect of introducing IHIH than an effect of ISO. Physical injury due to multiple intratracheal insertions could possibly explain a whole range of the DEGs and pathways found in this MS. In order to be really able to investigate the sole effect of ISO, experiments should be re-done in SHR rats receiving free, spontaneous inhalation with ISO compared to air only. Response: Regarding the issue of 'multiple' insertions, there are a total of 2, and these were spaced 24 hr apart. With regard to the earlier portion of this comment, please see above.
2) There is indeed evidence ISO inhalation results in altered cardiovascular function. Examples include concentration-dependent decreases in heart rate and blood pressure (PMID: 10622172) which may lead to more severe hypotension in hypertensive circumstances (PMID: 15574546). Others have also shown that vascular effects ex vivo and in vivo in hypertensive rats are more severe than in normotensive rats (PMID: 15574546). However, if cardiac gene expression is equally affected by ISO remains unknown. As outcomes in SHR are generally compared to e.g., WKY rats, the Authors should also consider taking into account the DEGs and pathways that RNA-seq yields for these animals on ISO vs. control. Response: It is unclear if the Reviewer is asking us to repeat these studies again but using WKY rats. If so, that is not possible. Further, use of other strains was criticized by the Study Section.
3) The methodological approach and pathway analyses based on the RNA-seq data looks sound. Response: Thank you.

Reviewer #2

This is a remarkable study, with timepoints reaching 360 days. It is indeed uncommon to see studies go into that level of depth with their measurements. It is yet more remarkable that there is any effect at this timepoint. This study is very intriguing and will undoubtedly be impactful in the field, and I congratulate the Authors on this monumental effort. However, I do think that several things need to be added to this paper to show individual gene measurements and plots of variance - for example showing some replicates for the later timepoints would reassure Readers that these timepoints are real measurements and provide more convincing evidence of these striking findings. Response: We thank the Reviewer for the very positive comments and encouragement. We agree that longitudinal plots to show variations across timepoints are useful and have added more of such plots.

I think this is a potentially very useful and illuminating study that should be published without delay. However, in its current state, it suffers from a lack of a few critical pieces that would make the study both easier to comprehend, more informative, and more convincing. I know there are a lot of comments here, but I hope these changes are ultimately easy to make and do not require a lot of extra work because it would be good to share this research with the community. Response: We thank the Reviewer for the suggestions. We have addressed all of the comments and incorporated all suggestions.

Major comments:

1. The most striking omission from this paper is the description in the results of any particular gene. This would help to clarify the main message of the paper and anchor the conclusions in more solid observations. GSEA can be misleading or confusing if not considered alongside the genes that are actually observed and I think more effort should be put into referencing some of the top significant genes in the body of the text and/or figures instead of only in the supplementary tables. Response: We agree that describing particular genes are of importance in general. GSEA can be misleading or confusing if not considered alongside the significant genes that are actually observed. As suggested, we have added tables that describe the specific genes in the important pathways. Given the relatively small sample size of rats at each timepoint and the large number of genes tested, we reported false discovery rates (FDR) in addition to unadjusted p-values. We observed a large number of DEG with low FDR that are less likely to be false positives; this provided a solid basis for the GSEA based functional pathway analyses. Also, individual DEG are more possibly false positives than DEG clustered in pathways; thus, we focus on reporting top DEG in the important pathways (see new Tables 2 and 3 for genes associated with short-term effects and new Tables 5 and 6 for genes associated with long-term effects. For example, DEG at Day 1, Bnip ($p = 8.40 \times 10^{-6}$, $FDR = 2.01 \times 10^{-2}$), Adam17 ($p = 2.88 \times 10^{-5}$, $FDR = 2.57 \times 10^{-2}$), Cx3cl1 ($p = 1.10 \times 10^{-4}$, $FDR = 4.33 \times 10^{-2}$) are in the response to wound pathways that are significant at Day 1. However, significance faded away after Day 30 (see Table 3). We have also added tables presenting significant genes with low FDR that are newly-manifested at late post-exposure timepoints in selected pathways. The existence of DEG with low FDR levels justifies the GSEA analyses. We have revised the MS accordingly.
2. There should be a statement about deposition of the FASTQ files into the SRA database with the BioProject accession number. Response: Yes, we plan to deposit data files into the SRA database after the MS is accepted for publication.
3. In the version of the MS that I downloaded, there appears to be no figure legends. I clarified with the editor that the short descriptions in the results are meant to be the figure legends. However,

it seems to me that there are only figure TITLEs here alongside a results section. A true figure legend would speed the comprehension of the data and should have information like N/timepoint, etc. For me it is very unconventional not to have a formal figure legend with descriptions of what is being plotted etc. Response: Figure legends have now been provided.

4. The title "Heatmaps for 212 selected genes impacted by ISO at each timepoint" is extremely confusing given that these heatmaps apparently comprise data that is the 87 up genes and the 125 down genes which are largely changing only at the Day 1 period. I might suggest Heatmaps following 212 DEGs from the Day1/Day30 timepoints over time" or something like that. Moreover, this is the only plot in the paper that shows variance, and it seems like there is a bit of an outlier. This is not necessarily a huge problem but it raises the question of where is the variance for the other gene measurements plotted and how carefully were outliers looked for. This should be shown somewhere. Response: Thank you for the suggestion. We have revised the title for the heatmap as suggested.

5. One solution to this is to show a PCA plot or to comment on whether there are outliers somewhere in the results even if you did not find any. Response: We agree that PCA plots are useful to identify possible outliers and informative patterns of clustering. We have conducted PCA and plotted the top two principal components. The PCA plot over different days reveals the non-supervised clustering of Naïve rats versus ISO treated rats based on the expressions of all candidate genes after filtering. The pattern of clustering of the two groups indicates that there is no evidence of outliers. We also looked for evidence of potential batch effect, but did not find evidence of batch effects (by plotting gene expressions of the same tissue that are repeatedly measured at different timepoints). This PCA plot has now been provided as Supplemental Figure 1.

6. RELATED to the earlier confusion, there apparently are other genes changing at the other timepoints at least from the volcano plots in the earlier figures. Despite this, these data are not really plotted or explained. The finding that seemingly hundreds of genes are modulated at day 360 is QUITE STRIKING and should be explained further. ALSO, the heatmap plots that allow for the evaluation of variance (i.e. how noisy is this signal) are REALLY VALUABLE for reviewing these data and would potentially make these data much more compelling and convincing. Response: We agree there are other genes changing at the other timepoints as can be seen from the volcano plots in the early figures. These differentially-expressed genes at Day 360 potentially reflect long-term impact of the ISO and may include both persistently significant DEGs and potentially newly-manifested DEGs at late timepoints. We also agree that the finding that seemingly hundreds of genes are modulated at Day 360 is quite striking and should be explained further. As suggested, we conducted further examination of these genes by investigating consistently differentially-expressed genes that are newly manifested only at late timepoints post-ISO exposure (at Days 240 and 360). We also conducted trend analysis as significant and consistent trends may indicate potentially long-lasting effects of ISO. Again, given the relatively small sample size of rats and the large number of genes tested, as in a typical multiple testing situation, a large portion of the genes with $p < 0.05$ are likely due to chance. Thus, we considered false discovery rates (FDR) in addition to unadjusted p-values. Also, individual DEG are more possibly false positives than groups of DEG clustered in important pathways.

As such, we focused on reporting particularly DEG in the important pathways in investigation of newly manifested DEG at the later timepoints (Days 240 and 360). We note there are a large number of DEG with $FDR < 0.01$. The very low FDR levels of many DEG suggest evidence of signals beyond chance. Similar to the examination of genes and pathways with short-term effect, we have added new longitudinal trend analyses of genes and pathways to establish evidence of

long-term effects. The newly added Tables 4-6 represent data from the investigation of genes that manifested only at late timepoints with long-term effect due to ISO (parallel to Tables 1-3 for DEGs with short-term effect).

Lastly, as suggested, we constructed extra heatmap plots over time on expressions of the genes that are consistently differential expressed at late timepoints (Days 240 and 360). More specifically, heatmaps were generated for the top 50 consistently up-/down-regulated DEG defined from Days 240 and 360 (new Figures 9 and 10). Figure 9 shows the top 50 up-regulated DEG were not up-regulated as a result of ISO until Days 240 and 360. Figure 10 shows that the top 50 down-regulated genes were barely down-regulated until Days 240 and 360. These new heatmaps provide useful insights, i.e., the volcano plots for Day 360 indicated a large number of differentially-expressed genes 1-yr after the exposure (i.e. reflecting long-term effects). The patterns in the new heatmaps were also supported by the longitudinal trajectory plots (new Figures 7A and 7B). In these figures, longitudinal line graphs for gene expression were also plotted; the mean line allows for the evaluation of the trend and variation of the DEG. Interestingly, as shown in the new Figure 8, these consistently differentially-expressed genes are enriched in multiple pathways, including oxidative phosphorylation, ribosome, angiogenesis, mitochondrial translation elongation, and other pathways.

7. RELATED to the above point, It would make sense to have plotted the top 50 or so genes for example for each timepoint in heatmap format first, and then shown the decay of the day1 genes over time subsequently. In fact, it seems that the decay of these genes is adequately presented in the line graphs, and does not really need to be shown again in the heatmaps. At first glance, it gave the impression that simply the genes detected at 360 days were just noise because it seemed like the heatmap for Day 360 was representing genes identified at this timepoint, and it was not intuitive that these corresponded to Day 1 genes being examined at Day 360. Overall, I would propose to make heatmaps of the significant genes for each time-point and perhaps move the other heatmaps either to the supplementary or simply show them after these other plots. Response: As suggested, we have plotted the top 50 genes for each timepoint in heatmap format (new Figure 2A and 2B). We are glad that the Reviewer found the decay of these genes with short-term effects is adequately presented in the line graphs. Similarly, for the newly-examined genes with long-term effects, we have plotted the consistently differentially-expressed genes at Day 360 in both heatmaps (using top 50 consistent DEG associated with long-term effects) for each timepoint (new Figures 8 and 9) We also now present longitudinal line graphs (new Figures 7A and 7B) to reflect increasing/decreasing trends.

Minor comments:

1. There is an overuse of hyphens, (for example Side-effects should not be hyphenated). Response: The MS has been revised to minimize use of hyphens.

2. In the first sentence ‘commonly-used’ and ‘widely-used’ are redundant descriptors. Response: The sentence has been modified. Thank you.

3. The sentence “Clearly, the potential for cardiovascular effects from repeated exposures to ISO should be carefully investigated” could be refined to contain more information. For example the previous paragraph sets up the idea of reducing cell injury, whereas this sentence is by comparison quite vague. Response: This sentence has been deleted and the opening of the paragraph modified to flow more smoothly into the subsequent content. We apologize for this confusing opening line.

4. In the results, the phrase "a vast number of DEG" could be replaced with the exact number up and down which would be more informative. Perhaps this number could also be incorporated into the figure itself for ease of reference. I recognize that this number does appear later in the text, but perhaps it should be a number first and a descriptor second? Alternatively, perhaps you could replace it with a relativistic statement (i.e. more than XYZ timepoint, etc.) Response: We agree and the sentence has been modified accordingly.

5. Specific information on library preparation should be provided - i.e. is it PolyA selection? The statement "(mainly including mRNA and long non-coding RNA)" is not sufficient. Response: More details have been added.

6. The color scale in Figure 6 could be explained using some kind of scale or written in the figure legend. It is apparent that the darker color corresponds to more significance but perhaps a flame scale here would be helpful. Response: Done as suggested.

7. Showing more longitudinal plots would be more concordant with the data structure but is not strictly necessary. Response: We have added more longitudinal plots as suggested.
