# Supplementary material for: Longitudinal impact on rat cardiac tissue transcriptomic profiles due to acute intratracheal inhalation exposures to isoflurane
Source: PLoS One. 2021 Oct 14;16(10):e0257241. doi: 10.1371/journal.pone.0257241 (PMC8516213; doi:10.1371/journal.pone.0257241)
Supplement: Attachment — Submitted filename: Response to Reviewers.pdf [file pone.0257241.s012.pdf]

PONE-D-21-09743R1
Longitudinal Impact on Rat Cardiac Tissue Transcriptomic Profiles due to Acute Intratracheal Inhalation Exposures to Isoflurane

Reviewer #1
Aim of the study described in the manuscript by Park et al. is to elucidate the effect of the inhalation anesthetic isoflurane (ISO) on gene expression patterns of cardiac tissue. Short- and long-term effects on gene regulation by ISO, especially in models of cardiovascular disease, remain largely unexplored. Park et al., investigated the role of ISO on SHR rats by 2 consecutive days of 2 hr ISO (2%) intra-tracheal inhalation (IHIH) vs. air only inhalation. Hearts were explanted on D1, D30, D240 and D360 post-exposure. Their main finding was that ISO exposure leads to changes in cardiac gene expression. In their revised manuscript, the author place more emphasis on acute versus (novel) long-term effects, hereby discerning the differential pathways induced by ISO exposure.

I appreciate the additional effort put in the revised MS, both in writing and data. The additional heatmaps, Volcano-plots and GO-pathway analysis give a more in-depth overview of the RNA-seq data. Please find a more detailed overview of my comments below.

1) Regarding the experimental set-up: the authors have shared their thoughts and considerations on choosing this specific approach (eg. IHIH vs free-breathing). I would suggest to include part of this information in the Materials and Methods sections and discussion. This can be done to both emphasize and discuss the differences in acute (e.g. D240) DEG patterns. Response: We thank the Reviewer for the comments. We have included part of this information in the revised Materials and Methods and Discussion sections.

2) In general, the Authors have made a clearer distinction in representing and discussing the acute and long-term DEG patterns. This gives the reader a better insight between the types of pathways regulated. Indeed, I believe the long-term, newly manifested, effects are the most interesting; regulation of angiogenesis, extracellular matrix organization, (OXPHOS and FA) metabolism and cell signaling. While I understand that the authors will dedicate a separate manuscript on how these changes may lead to heart function alterations, lacks information on how the SHR model relates to previous work in healthy animals, which links to point 3) and the question of adding a WKY control. If the authors primary goal is to share information on routine ISO employment in a variety of clinical settings, patients at risk should be identified. Now all previous models and spatio-temporal changes are equally discussed. Response: We thank the Reviewer. We now discuss previous works in healthy animals showing ISO effects in host heart tissues. These cited findings align with some of the data obtained here showing that genes for a number of the components within these pathways were impacted in a time-related manner in ISO-exposed SHR hosts. Furthermore, Kouki at al. (2016) reported isoflurane-induced cardiorespiratory effects between rat strains. They showed that SHR rats are more sensitive to suppressive effects of isoflurane on cardiovascular function among rat strains (SHR, WKY, and SD rats). Therefore, DEG patterns in healthy animals may be different in SHR. We added this information in the Discussion section. We may address the comparison of DEG patterns between WKY control and SHR in the future study.

3) The Discussion lacks connections between existing literature and the current study on potential mechanisms of ISO to induce these DEG patterns. Specifically by now separating the acute and long-term phase, some explanation should be included how these transient changes come into play. Response: We found that the acute ISO effect was primarily upon genes/pathways associated with wounding, local immune function, inflammation, and circadian rhythm regulation in the heart of the SHR rats, and that the novel long-term ISO effects were upon genes/pathways associated with oxidative phosphorylation, ribosome, angiogenesis, mitochondrial translation elongation, and focal adhesion. How these changes come into play at each stage post-exposure is still unknown. Discerning how each may/may not play a role in actual physiologic changes in the heart tissues is the subject of an ongoing study.

4) Novel text addition lines 531-536 seems to have overlapping message with lines 537-541. The distinction between these statements is better described in line 604-611. Response: We have re-written lines 531-541 to provide a clearer distinction between these statements.

Reviewer #2

This is an interesting work. Overall I support the publication of these data as an important addition to the scientific record. Please see my attached detailed comments for minor revisions to improve the communication of these findings to the reader and clarify several issues I feel may be important for the reader.

The changes made to the paper greatly strengthen the results. The inclusion of specific genes in the tables as well as narratively in the results greatly improves the experience as a reader. Particularly, I greatly appreciated the inclusion of the section titled "Significantly expressed genes newly-manifested at late timepoints…" which greatly expanded the comprehension and clarity of the manuscript.

The MS could still benefit from careful proofreading as there are a variety of minor grammatical errors throughout the highlighted changes that can be distracting while reading the paper.

Major comments:

1) While some of the comments of the other reviewer are beyond my level of expertise, perhaps a diagram showing what ITIH means and the experimental groups would be helpful to educate the reader on why you designed the study this way and why you believe this procedure is not the cause of your observations. I feel that a diagram to explain the ITIH time points and what the procedure looks like would be clarifying for the reader. I.e., isn't the ITIH catheter quite physically far from the heart? Some of these technical nuances could be directly mentioned or explained in narrative form in the "Experimental design" section to make it clear to the reader why that design was selected. This would be a good opportunity to highlight the rationale of the study and explain the choice of control groups further. While I cannot expressly comment on the need or lack of need for additional experiments I do feel this could be directly addressed in part by expanding this section with more rationale. In a sense, it does seem rather unlikely that ITIH causes changes in transcriptional signature 360 days out, but it's also not entirely apparent why isoflurane would cause these changes. I.e. is this really the interaction of the model with the isoflurane? Maybe this clarification would

make the claims of the paper more believable as I can understand how they can seem quite outlandish if not carefully articulated (major gene changes 1 year later…) Response: We thank the Reviewer. As a diagram describing ITIH is well-presented in a paper by Rivard at al. (2006), we do not believe the same system diagram should be included here. However, to help provide clarity as to why the specific experimental design employed here was used, additional background information for why effects from ISO had to be analyzed (and its means of delivery) are now provided for the Readers.

2) Perhaps the authors could clarify if they would expect any effects in healthy animals and speculate on whether this is an interaction between the model and the drug? Response: Kouki at al. (2016) reported ISO-induced cardiorespiratory effects could differ between rat strains. For example, they showed that SHR rats were more sensitive to suppressive effects of ISO on cardiovascular function when compared to WKY and SD rats. Thus, DEG patterns in healthy animals may be different in SHR than in those other strains. We added this information in the Discussion section. We will address comparisons of DEG patterns arising from ISO exposure between healthy WKY and SD rats and SHR in a future study.

3) The new MS is vastly improved in terms of capacity to read and comprehend. Alongside this improvement, there are some additional questions that now arise as I can begin to think more about what is being presented. Is it perhaps beneficial to mention that mutations in COL4A2 and COL4A1 have been linked to heart disease? These seem to be major structural proteins that are altered in this model suggesting rather dramatic effects. Response: We thank the Reviewer for the suggestion. Indeed, several publications showed that mutations in COL4A1 and COL4A2 are linked to heart diseases. We discuss this further in the revised Discussion.

4) I am a bit fuzzy on the experimental design in one key element. This is another area where a diagrammatic overview would be useful. It seems from the PCA plot that naïve animals were followed over time and collected at 1 day / 30 day etc. to control for aging and disease progression in the SHR model, but this is really not explicitly stated in the manuscript. Without careful reading, it is possible to misinterpret these findings as being age-related, and I think the authors should carefully explain that the naïve animals were collected in an age-matched fashion and not simply the same time zero time point used to compare to each later time point. Additionally, this could be conveyed by listing the total number of animals which seems to be in the realm of ~50 animals, which is quite large for an RNA-Seq study and emphasizes how much time and resources was put into this study. Response: Naïve rats were collected in an age-matched fashion. We have added this to the "Experimental Design".

5) Correspondingly, can the authors comment on whether the controls also showed induction of Col4a2 and Col4a1 over time? I.e., is that the effect of the SHR model over time on the heart function? And is that in fact exacerbated by your treatment with isoflurane? I would understand if the "control time course of this model" is in a way a separate study, but perhaps we need to understand that to contextualize these results a bit more? Could the authors comment if this will be an additional study or clarify this in the discussion somewhere? Since the data are already extant it is something that I wondered when reading this. Response: Yes, we plan to submit a separate paper describing any age-related effects in the SHR model [over time] in a future study. As such, we do not include this type of information in the current MS.

6) If it isn't going to be in a future study, I would include a comparison between the early time point and day 360 just for the naïve animals SHR to understand what's going on in the model without any intervention. Response: Yes, we plan to submit a separate paper on age-related effects on the SHR in a future study. Therefore, we do not include this in the current MS.

7) As an additional commentary on the above, it would be interesting to have done histology on these sections and I find it a bit striking that you opted not to. While I don't think this is a necessary component, I would suggest at least mentioning in the discussion that histological analyses would be useful in the future to understand what kind of tissue remodeling is going on, etc. that would lead to these changes. Response: This is included in revised Discussion.

8) I am unsure about the authors choice of the phrase "Newly manifest", which is used as a verb in several places. I would recommend to replace all instances of the term "newly manifest" with "delayed onset" or "late onset" or something more conventional. This is becoming a major focus of the study, and it obscures the clarity of the central message. Response: We have replaced the phrase "newly manifest" to "late onset" throughout the text.

9) The BioProject number can be generated now before uploading the FASTQ files and included in the revision of the manuscript. It is advantageous to have this BioProject number in the manuscript itself as it will help link the deposited dataset to your paper which will enhance its utility for the field. Response: We are happy to share data and we will generate a BioProject number and upload the FASTQ files when the MS is accepted.

Minor comments:
The reviewer recommends careful reading for grammar at this stage.

Abstract
1) I believe "total RNA extracted for…" should be "total RNA was? extracted" Response: The sentence was modified.
2) The phrase "tissue (damage) healing" is quite bizarre. Can something clearer be substituted here? Response: The text was modified.

Manuscript
1) I have generally encountered that people pluralize DEG as DEGs although if you would prefer it the way it is, it is not necessary to change. Response: The Authors would prefer DEG.

2) Page 4, line 81, the expansion of differentially-regulated genes (DEG) is incorrect and should read "differentially expressed genes (DEGs)." Response: The sentence was modified.

3) Page 4, line 74-76 "it is also not certain if such repeated exposures to ISO can also lead to …" one of the "also" should be deleted. Response: One "also" was deleted.

   a. AND "repeated acute exposure to ISO influence gene" should be "influences"? Or possible "exposures". Response: The text was changed.

   b. Line 86, delete ", here," Response: It was deleted.

   c. Line 88, should be "received" to match the past tense used throughout the paragraph. Response: The text was fixed.

4) Page 7. Oligo misspelled as "Ologo." Response: The text was changed.

5) Page 9. "The longitudinal trajectory of differential expression was plotted based on by log fold-change (logFC) at each post-exposure timepoint for each gene to examine short-term or long-term trends." This sentence should be revised. "on by" is a typo AND in general this sentence does not read well. Response: The text was revised. Thank you.

a. A statement should be added to the end of the sentence to explain the usage of the Joncheere-Tepstra test. … "was tested using J-T test to… ". Optionally, at other points when this test is used it may also be useful to reprise the rationale of this test, as it is somewhat uncommon for the lay reader. Response: A rationale statement for the test was added to the Revised Methods as well as at other points in the revised Results.

6) Page 23. "The significance of these genes didn't appear (significantly) until day 240". This sentence must be revised. Response: The text was revised.

7) Page 25. "Overtime" is not the same as "over time". This should be revised. Also, Line 462: the word "barely" is not informative and could be replaced. Response: The text was changed.
