# Supplementary material for: Longitudinal impact on rat cardiac tissue transcriptomic profiles due to acute intratracheal inhalation exposures to isoflurane
Source: PLoS One. 2021 Oct 14;16(10):e0257241. doi: 10.1371/journal.pone.0257241 (PMC8516213; doi:10.1371/journal.pone.0257241)
Supplement: Attachment — Submitted filename: Response to Reviewers.pdf [file pone.0257241.s013.pdf]

PONE-D-21-09743R2
Longitudinal Impact on Rat Cardiac Tissue Transcriptomic Profiles due to Acute Intratracheal Inhalation Exposures to Isoflurane
PLOS ONE

Reviewer #1
Aim of the study described in the manuscript by Park et al., is to elucidate the effect of the inhalation anesthetic isoflurane (ISO) on gene expression patterns of cardiac tissue. Short- and long-term effects on gene regulation by ISO, especially in models of cardiovascular disease, remain largely unexplored.
The additional changes from this second round have further improved the content and message of the manuscript. My comments therefore primarily concern writing to improve clarity and readability.

1) Line 122 - 125 repetition of materials and methods with lines 136-137 but final concentration of ISO in not consistent (2% vs 2.5%) Response: We thank the Reviewer for the comment. The concentration was corrected to 2.5% in the entire manuscript.

2) It is common that the total amount of animals, e.g., animals per timepoint per group, are described in the Materials and Methods (line 114 onwards) and not just the figure legends. I assume the Figure legend 1A means n = 6 or n = 5-6 animals per group PER TIMEPOINT. Ideally, use of animals is repeated for each Figure. Response: The animal numbers used has been added in the Materials and Methods (line 114), as well as in each figure legend. These values are per group per timepoint.

3) Materials and methods from line 126: text explaining the use of ITIH because of the WTC-dust experiments confuses me. Do the Authors mean that SHR animals on ITIH were part of a control for WTC-dust trials? I think it must be explicitly mentioned that the animals in this manuscript were not exposed to WTC-dust themselves. Response: This clarifying text has been added.

4) Caption Table 6, Line 463; addition of 'Late onset' DEG Response: The text was added.

5) Line 562-553: 'Kouki et al. (2016) reported strain-specific isoflurane (…)' Response: The text was modified.

6) Line 639-640: missing references about comparisons to other inhalant anesthetics Response: The missing references have been added.

7) Line 654: Copy-paste of response to reviewer. I would remove 'respectfully' Response: The text was modified.

8) Line 659: Copy-paste of response to reviewer. I would remove ' -and still do-' and change to 'Thus we believe' Response: As lines 657-664 were deleted (see comment #9), the text was deleted.

9) Insertion of lines 657-664 largely overlapping in content with following lines 667-673. Response: See above.

Reviewer #2
I have no further revisions. I recommend the publication of this manuscript if there are no other comments from other reviewers or the editor. Response: Thank you.

Journal Requirements:
Please review your reference list to ensure that it is complete and correct. If you have cited papers that have been retracted, please include the rationale for doing so in the manuscript text, or remove these references and replace them with relevant current references. Any changes to the reference list should be mentioned in the rebuttal letter that accompanies your revised manuscript. If you need to cite a retracted article, indicate the article's retracted status in the References list and also include a citation and full reference for the retraction notice. Response: New references (#70 and 71) were added in this revised manuscript.
