# Supplementary material for: Longitudinal impact on rat cardiac tissue transcriptomic profiles due to acute intratracheal inhalation exposures to isoflurane
Source: PLoS One. 2021 Oct 14;16(10):e0257241. doi: 10.1371/journal.pone.0257241 (PMC8516213; doi:10.1371/journal.pone.0257241)
Supplement: Attachment — Submitted filename: comments.docx [file pone.0257241.s010.docx]

Title: Longitudinal Impact on Rat Cardiac Tissue Transcriptomic Profiles due to Acute

Intratracheal Inhalation Exposures to Isoflurane

This is a remarkable study, with time points reaching 360 days. It is indeed uncommon to see studies go into that level of depth with their measurements. It is yet more remarkable that there is any effect at this time point. This study is very intriguing and will undoubtedly be impactful in the field, and I congratulate the authors on this monumental effort. However, I do think that several things need to be added to this paper to show individual gene measurements and plots of variance – for example showing some replicates for the later time points would reassure readers that these time points are ***real*** measurements and provide more convincing evidence of these striking findings.

I think this is a potentially very useful and illuminating study that should be published without delay. However, in its current state it suffers from a lack of a few critical pieces that would make the study both easier to comprehend, more informative, and more convincing.

I know there are a lot of comments here but I hope these changes are ultimately easy to make and do not require a lot of extra work because it would be good to share this research with the community.

Major comments:

1. The most striking omission from this paper is the description in the results of any particular gene. This would help to clarify the main message of the paper and achor the conclusions in more solid observations. GSEA can be misleading or confusing if not considered alongside the genes that are actually observed and I think more effort should be put into referencing some of the top signficiant genes in the body of the text and/or figures instead of only in the supplementary tables.
2. There should be a statement about deposition of the FASTQ files into the SRA database with the BioProject accession number.
3. In the version of the manuscript that I downloaded there appears to be no figure legends. I clarified with the editor that the short descriptions in the results are meant to be the figure legends. However, it seems to me that there are only figure TITLEs here alongside a results section. A true figure legend would speed the comprehension of the data and should have information like N per time point, etc. For me it is very unconventional not to have a formal figure legend with descriptions of what is being plotted etc.
4. The title “Heatmaps for 212 selected genes impacted by ISO at each timepoint” is extremely confusing given that these heatmaps apparently comprise data that is the 87 up genes and the 125 down genes which are largely changing only at the day1 period. I might suggest Heatmaps following 212 DEGs from the Day1/Day30 time points over time” or something like that. Moreover, this is the only plot in the paper that shows variance, and it seems like there is a bit of an outlier. This is not necessarily a huge problem but it raises the question of where is the variance for the other gene measurements plotted and how carefully were outliers looked for. This should be shown somewhere.
5. One solution to this is to show a PCA plot or to comment on whether there are outliers somewhere in the results even if you did not find any.
6. RELATED to the earlier confusion, there apparently are other genes changing at the other time points at least from the volcano plots in the earlier figures. Despite this, these data are not really plotted or explained. The finding that seemingly hundreds of genes are modulated at day 360 is QUITE STRIKING and should be explained further. ALSO, the heatmap plots that allow for the evaluation of variance (i.e. how noisy is this signal) are REALLY VALUABLE for reviewing these data and would potentially make these data much more compelling and convincing.
7. RELATED to the above point, It would make sense to have plotted the top 50 or so genes for example for each time point in heatmap format first, and then shown the decay of the day1 genes over time subsequently. In fact, it seems that the decay of these genes is adequately presented in the line graphs, and does not really need to be shown again in the heatmaps. At first glance, it gave the impression that simply the genes detected at 360 days were just noise because it seemed like the heatmap for day360 was representing genes identified at this time point, and it was not intuitive that these corresponded to day 1 genes being examined at day 360. Overall, I would propose to make heatmaps of the significant genes for each time point, and perhaps move the other heatmaps either to the supplementary or simply show them after these other plots.

Minor comments:

1. There is an overuse of hyphens, (for example Side-effects should not be hyphenated).
2. In the first sentence commonly-used and widely-used are redundant descriptors of isoflurane.
3. The sentence “Clearly, the potential for cardiovascular effects from repeated exposures to ISO should be carefully investigated” could be refined to contain more information. For example the previous paragraph sets up the idea of reducing cell injury, whereas this sentence is by comparison quite vague.
4. In the results, the phrase “a vast number of DEG” could be replaced with the exact number up and down which would be more informative. Perhaps this number could also be incorporated into the figure itself for ease of reference. I recognize that this number does appear later in the text, but perhaps it should be a number first and a descriptor second? Alternatively, perhaps you could replace it with a relativistic statement (i.e. more than XYZ time point, etc.)
5. Specific information on library preparation should be provided – i.e. is it PolyA selection? The statement “(mainly including mRNA and long non-coding RNA)” is not sufficient.
6. The color scale in figure 6 could be explained using some kind of scale or written in the figure legend. It is apparent that the darker color corresponds to more significance but perhaps a flame scale here would be helpful.
7. Showing more longitudinal plots would be more concordant with the data structure but is not strictly necessary.
